# Quasi-phase-matched laser wakefield acceleration of electrons in an axially density-modulated plasma channel

**DOI:** 10.1038/s41598-021-94751-y

**Published:** 2021-07-26

**Authors:** M. Sedaghat, S. Barzegar, A. R. Niknam

**Affiliations:** Laser and Plasma Research Institute, Shahid Beheshti University, 1983969411 Tehran, Iran

**Keywords:** Plasma physics, Plasma-based accelerators

## Abstract

Quasi-phase matching in corrugated plasma channels has been proposed as a way to overcome the dephasing limitation in laser wakefield accelerators. In this study, the phase-lock dynamics of a relatively long electron bunch injected in an axially-modulated plasma waveguide is investigated by performing particle simulations. The main objective here is to obtain a better understanding of how the transverse and longitudinal components of the wakefield as well as the initial properties of the beam affect its evolution and qualities. The results indicate that the modulation of the electron beam generates trains of electron microbunches. It is shown that increasing the initial energy of the electron beam leads to a reduction in its final energy spread and produces a more collimated electron bunch. For larger bunch diameters, the final emittance of the electron beam increases due to the stronger experienced transverse forces and the larger diameter itself. Increasing the laser power improves the maximum energy gain of the electron beam. However, the stronger generated focusing and defocusing fields degrade the collimation of the bunch.

## Introduction

Since the concept of laser-wakefield accelerators (LWFAs) was proposed^1^, tremendous progress has been made in the field of “advanced accelerators”. This smaller-scale technology is capable of accelerating electrons to 1 GeV in a few centimeters^2–6^. In LWFAs, the ponderomotive force of the laser pulse expels the background plasma electrons and excites a trailing plasma wave behind the driver^7,8^. For many applications such as high energy physics experiments^9,10^, advanced light sources^11,12^, and compact FELs^13^, bunch quality and energy are very important factors. It is needless to say that, in order to produce electron beams with appropriate qualities, studying various accelerator structures via experiments and simulations is indispensable.

To successively accelerate an electron beam to higher energies, it is required to overcome some major limitations, i.e. dephasing, pump depletion, and diffraction^6,9,14,15^. The dephasing length is the characteristic length for a relativistic electron to outrun the accelerating region of the wakefield, which scales as $L_{\rm {d}} \propto n_{0}^{-3/2}$^15^, where $n_{0}$ is the plasma density. Since the maximum accelerating field scales as $E_{\rm {max}} \propto n_{0}^{1/2}$, increasing the dephasing length by decreasing the plasma density leads to an increase in the maximum energy gain, $\Delta \gamma \propto E_{\rm {max}} L_{\rm {d}} \propto n_{0}^{-1}$. The laser pump depletion length, $L_{\rm {dep}}$, scales with the plasma density in the same way as the dephasing length does^15^. Thus, by lowering the plasma density, the depletion limitation can be overcome, and, at the same time, higher energy gains can be achieved. However, a lower plasma density means a longer acceleration distance, and propagating the laser pulse over a long distance is challenging due to diffraction^9^. Self-focusing of the laser pulse may provide a solution. When the laser power *P* is well above the critical power, $P_{\rm {crit}}=17{\omega _{0}}^2/{\omega _{\rm {p}}}^2\,\hbox {GW}$, where $\omega _{0}$ and $\omega _{\rm {p}}$ are the laser frequency and plasma frequency respectively, a deep channel is produced by the ponderomotive pressure of the intense short laser pulse, and the pulse can be self-guided through the whole acceleration length^2,16^. However, this solution has its own downside: At low plasma density, $P_{\rm {crit}}$ is large, and self-focusing does not occur unless the pulse power is high^15,17,18^. Although petawatt class laser systems provide high intensity pulses, the rate of etching and pump depletion is faster at high intensities^15,19^. In addition, the low repetition rate ($\le 10\,\hbox {Hz}$) of high power laser systems limits the performance and attractiveness of ordinary LWFA technique for some applications such as laser-plasma X-ray sources, in which high average output is desirable^20^. Diffraction can also be reduced by increasing the Rayleigh length via using a larger laser spot size, but this approach requires high pulse power too. Therefore, in order to extend the acceleration length for a laser pulse of moderate power, using a preformed plasma channel^21,22^ is necessary. A plasma channel is able to guide a pulse over long distances regardless of its power^2,23^.

Even though multiple methods are already proposed in order to achieve higher electron energy gain by extending $L_{\rm {d}}$, such as tapering the plasma density profile within a plasma waveguide^21,24–27^ and electron beam-laser pulse synergy^28,29^, dephasing still remains as an essential limitation in these approaches. Using multiple LWFA stages can overcome the dephasing limitation, but successively accelerating an electron beam to higher energies is a challenging approach. Transferring a beam to the next plasma stage^30–32^ can dramatically damage its quality and leads to charge loss. Axially corrugated plasma channel has been suggested as a means for eliminating the dephasing limitation for both DLA and LWFA^33–37^. This kind of channel has been successfully generated by Layer et al. with two methods^38,39^. In the first method, a channel-generating laser pulse is radially modulated with the help of a “ring grating” (RG), then is focused onto a gas jet by an axicon, thus the pattern produced by the RG is mapped onto the optical axis, generating an axially modulated plasma. An RG is not used in the second method. Instead, the target cluster jet is modulated by placing a grating composed of parallel wires at the orifice of the cluster jet. An ionizing laser pulse which comes out of an axicon passes through this structure and the corrugated channel is produced. For a pulse propagating in a corrugated channel, the guided mode is composed of spatial harmonics whose associated phase velocities can be tuned by modifying the modulation period^34,36,37^. Using this structure for LWFAs, the phase velocity of the plasma wave undergoes oscillations. Consequently, the plasma wave itself is composed of spatial harmonics. This scheme can be used in LWFA based on quasi-phase-matching (QPM-LWFA)^37^, in which the phase velocity of one of the spatial harmonics is matched to the velocity of the accelerated electron beam, which is almost equal to the speed of light in vacuum. For a plasma channel, this can be achieved by matching the density modulation period of the corrugated channel and the dephasing length. As a result, the relativistic electrons experience a near-constant axial acceleration from the selected spatial harmonic, while the time average of the acceleration of all other spatial harmonics is zero. This scheme allows the electrons to gain energy over a distance much longer than the dephasing length. The QPM method improves the acceleration efficiency at low pulse powers and provides an appropriate guiding structure for pulse propagation. As we already mentioned, for some applications of LWFA, such as advanced X-ray radiography, use of lower power pulses is advantageous. High-peak-power laser systems typically used in LWFA require low frequency flash lamps for pumping. This limits the repetition rate of such laser systems to $\sim$1–10 Hz, leading to low average x-ray brightness. Understanding this issue motivates the development of novel laser-driven acceleration methods that can be conducted with a relatively low laser peak power ($<1\,\hbox {TW}$) and high repetition rates. There is therefore considerable interest in using lower peak power lasers with higher repetition rates to accelerate relativistic electrons^40–42^.

In this paper, the dynamics of externally injected electron bunches is studied in QPM-LWFAs via fully self-consistent two-dimensional (2D) particle-in-cell (PIC) simulations. A density-modulated plasma channel has a significant influence on the final features of the accelerated bunch. The energy spectrum, trace space, emittance, and electron distribution are analyzed through the bunch propagation in the corrugated plasma channel. The effect of the initial electron beam energy, the beam density, its diameter, the length of the waveguide, and the pulse power on the transverse properties of the bunch is discussed. It is observed that, by choosing appropriate conditions, it is possible to generate a monoenergetic electron beam with rms energy spread of $1.6\%$ in QPM-LWFA. It is shown that, for optimum bunch charge, the beam remains collimated for a longer propagation distance. The self-field of the bunch superimposes with the laser-induced wakefield and hinders the defocusing of the beam. Our simulations show that higher initial energy for the bunch leads to significant emittance growth due to faster growth in the variance of particles momenta in the transverse direction. We observe that the injection of a bunch with a lower diameter can improve the bunch emittance because the transverse wakefield increases with the radial position and also the variance of the radial positions is larger for higher transverse beam size.

## Simulation model and parameters

**Figure 1:**
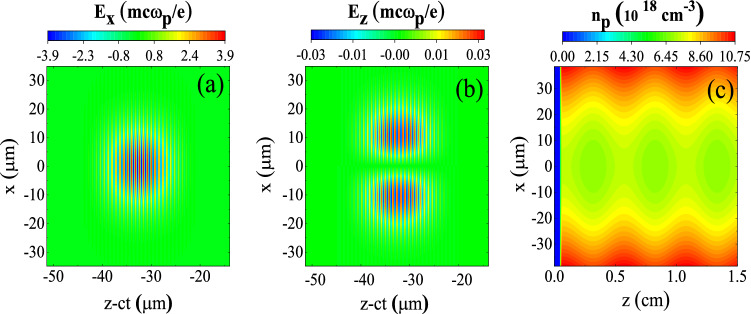
Heatmaps displaying **(a,b)** the transverse and axial components of the electric field $E_x$ and $E_z$ of a 30 fs, 0.5 TW, linearly polarized laser pulse with a spot size of $w_{0} = 15\,\mu \hbox {m}$, and **(c)** the plasma density of the corrugated plasma channel as a function of the transverse and axial coordinates, *x* and *z*.

To study the evolution and acceleration of externally injected electron beams of moderate energies in a corrugated plasma channel in the framework of QPM-LWFA, we have performed 2D particle-in-cell (PIC) simulations using the code OSIRIS^43^. In all simulations, we use a linearly polarized pulse of wavelength $\lambda _{\rm {L}}=0.8\,\upmu \hbox {m}$ with a sine-squared temporal profile and a Gaussian transverse profile which is focused into a waist radius of $w_0=15\,\upmu \hbox {m}$. Unless otherwise stated, the magnitude of the normalized vector potential $a_0\equiv {eA}/{m_{\rm {e}}c^2}$ is set to 0.25, corresponding to a low peak power of 0.5 TW and pulse energy of only 14 mJ. Here, $m_{\rm {e}}$ and *e* denote the electron rest mass and charge, respectively, and *c* is the speed of light in vacuum. The pulse duration is $\sigma _{\rm {FWHM}}=30\,\hbox {fs}$, and since in this work the on-axis period of plasma oscillations approximately equals $2\pi /\omega _{\rm {p0}}=42\,\hbox {fs}$, the chosen value for pulse duration is close to its optimal value for having the maximum wakefield amplitude^2^. Here, $\omega _{\rm {p0}} =(4 \pi e^{2}n_{\rm {p0}}/ m_{\rm {e}})^{1/2}$, in which $n_{\rm {p0}}$ is the average on-axis plasma density. In Fig. 1a,b, the normalized transverse $E_x$ and longitudinal $E_z$ field components of the laser pulse at time $\mathrm {t}=0$ are shown. The wakefield is normalized to the wave-breaking field $E _{\rm {WB}} = m_{\rm {e}} \omega _{p0}c/e$, which in our case, is approximately 254 GV/m.

The target plasma is a corrugated channel as shown in Fig. 1c. The vacuum region is followed by a pre-ionized density ramp of length $L_{\rm {ramp}}=200\,\upmu \hbox {m}$. The plasma channel starts right after the density ramp and its plasma density varies sinusoidally and parabolically in the axial and transverse directions, respectively:

1 $$\begin{aligned} n_{\rm {p}}(x,z)=n_{\rm {p0}}[1+\delta \sin (k_{\rm {m}}z)](1+n''_0 x^{2}/2), \end{aligned}$$

where $\delta$ is the modulation amplitude, $k_{\rm {m}}=2\pi /\lambda _{\rm {m}}$ is the wavenumber related to the modulation period $\lambda _{\rm {m}}$, and $n''_0$ determines the curvature of the plasma channel, which has the relation $n''_0=2 c^2 m_{\rm {e}} / \pi e^2 n_{\rm {p0}} w^4_{\rm {ch}}$ with the so-called “channel radius”, $w_{\rm {ch}}$. For optimal pulse propagation, $w_{\rm {ch}}$ must be equal to the waist radius of the pulse, $w_0$. It should be noted that without applying a plasma channel it is not possible to guide a low power laser pulse over long distances. The values of the parameters for the density profile are $n_{\rm {p0}}=7 \times 10^{18}\,{\rm cm}^{-3}$, $\delta =0.04$, and $\lambda _{\rm {m}}=5\,\hbox {mm}$. This value for $\lambda _{\rm {m}}$ is equal to the theoretical dephasing length of the waveguide, $L_{{\mathrm {d}}}=2\lambda _{\rm {p0}}^{3}\lambda _{\rm {L}}^{-2}(1+8/k_{\rm {p0}}^{2}w_{\rm {ch}}^2)$, where $k_{\rm {p0}}=\omega _{\rm {p0}}/c$ and $\lambda _{\rm {p0}}=2\pi /k_{\rm {p0}}$. When $\lambda _{\rm {m}}=L_{\rm {d}}$, the phase velocity of the $n=-1$ spatial harmonic is almost equal to the speed of the relativistic electrons. Thereby, the bunch can be accelerated by this individual spatial harmonic over a distance much longer than the dephasing length^37^. Although QPM increases the energy gain of the linear regime considerably, the energy gain is ultimately limited by some factors. First, the pulse propagation is not without energy loss. So the pulse propagation length is restricted to the pulse depletion length, $L_{\rm {dep}}\sim 4L_{{\mathrm {d}}}a_{0}^{-2}$^2^. Second, the changes in the shape of the pulse due to spectral redshifting can affect its wakefield. Third, the phase velocity of the $n=-1$ spatial harmonic is different from the velocity of its envelope^37^. While the acceleration phase of a relativistic electron with respect to this spatial harmonic does not change considerably, the electron actually does move relative to its envelope. Only as long as the electron is close to the peak of the envelope, it can experience a large acceleration.

At $\mathrm {t}=0$, the front edge of the laser pulse is located at the beginning of the ramp and a monoenergetic bi-Gaussian electron bunch is placed in the vacuum region. To maximize the energy gain, the beam is initially located $200\,\upmu \hbox {m}$ behind the peak of the laser pulse, coinciding with the peak of the moving envelope of the $n=-1$ spatial harmonic. Unless otherwise stated, the electron beam is initialized with the following parameters. The density profile is $n_{\rm {b}}=n_{\rm {b0}}\exp (-x^2/2\sigma _x^2)\exp (-z^2/2\sigma _z^2)$, in which the transverse and longitudinal radii are $\sigma _{x}=4\,\upmu \hbox {m}$ and $\sigma _{z}=8\,\upmu \hbox {m}$, respectively, and the peak density is chosen to be $n_{\rm {b0}}=3.5 \times 10^{16}\,\mathrm {cm}^{-3}$, giving a bunch charge of $q_{\rm {b}}=11\,\hbox {pC}$. The beam has a longitudinal momentum of $P_{z}/m_{\rm {e}}c=30$, corresponding to initial energy of 14.8 MeV, which is higher than the trapping threshold energy for $a_{0}=0.25$.

The simulations have been performed in a moving frame co-propagating with the laser pulse at the speed of light in vacuum, *c*. The size of the simulation box is 438 $\upmu$m $\times$ 77 $\upmu$m with 16,384 $\times$ 512 cells in the longitudinal and transverse directions. Absorbing boundary conditions are applied at the two longitudinal boundaries for the electromagnetic fields and for the particles.

## Results

When deriving quantities and distributions such as maximum energy, emittance, energy spectrum, etc. from the outputs of the simulations, we only take into account the electrons which are at a maximum distance of $15\,\upmu \hbox {m}$ from the axis, unless otherwise stated. We refer to the region defined by this condition as “the region-of-interest” (ROI). One of the quantities reported frequently in this section is the normalized rms emittance in the *x*-direction defined by

2 $$\begin{aligned} \epsilon _{\rm {N},x}=\frac{1}{m_0 c}\sqrt{\mathrm {var}(x) \mathrm {var}(P_x)-(\mathrm {cov}(x,P_x))^2}, \end{aligned}$$

in which $\mathrm {var}(x)$ and $\mathrm {var}(P_x)$ are the variances of the components of the positions and momenta in the transverse direction, and $\mathrm {cov}(x,P_x)$ represents the covariance of these two variables.

**Figure 2:**
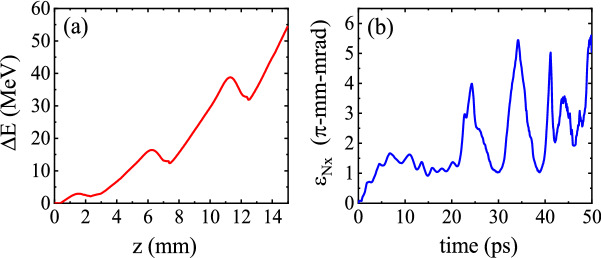
**(a)** Maximum energy gain $\Delta E$ of an electron bunch with an initial energy of $E_{0}=14.8\,\hbox {MeV}$ injected in the wakefield of a 30 fs laser pulse with $a_0= 0.25$ and $w_{0}$ = 15 $\mu$m, plotted versus the acceleration distance, and **(b)** bunch emittance $\epsilon _{\rm {N},x}$ as a function of the propagation time.

The variations of the maximum energy gain, $\Delta E$, as a function of the longitudinal position *z* for an electron beam injected from the optimal initial position are shown in Fig. a. It can be seen that the maximum energy gain of the electron beam reaches $\Delta E=54\,\hbox {MeV}$ after 1.5 cm of acceleration along the corrugated channel, which is close to the theoretical value. As Fig. 2b shows, the transverse bunch emittance $\epsilon _{\rm {N},x}$ increases with time. So, although a higher maximum energy gain can be obtained by extending the channel length, the transverse characteristics of the bunch can be degraded due to the defocusing of a large fraction of the bunch electrons.

**Figure 3:**
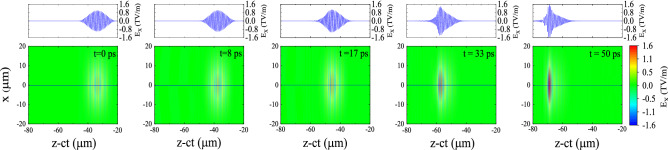
Snapshots showing the variations in the transverse field of the laser pulse (lower panel), $E_x$, and its on-axis line-out (upper panel) at different times in the corrugated plasma channel. The pulse initially has a peak amplitude of $a_{0} = 0.25$, a duration of 30 fs, and a spot size of $w_{0} = 15\,\upmu \hbox {m}$.

Figure 3 shows the nonlinear evolution of the femtosecond laser pulse propagating in the corrugated plasma channel. In this figure, the transeverse field of the laser pulse, $E_x$, and its line-out at the start of the simulation and also four axial distances of 2.4 mm (8 ps), 5.2 mm (17 ps), 1 cm (33 ps), and 1.5 cm (50 ps) are illustrated for an initial peak amplitude of $a_{0}=0.25$. As can be seen in this figure, the pulse is asymmetrically compressed and its maximum electric field is increased. The longitudinal compression of the pulse can be understood using the concept of “photon deceleration” in kinetic description of photons in plasma physics. Due to the ponderomotive force of the pulse, a local nonlinear gradient in the electron density is formed. Therefore, there is a gradient in the refractive index, with the refractive index being lower at the front of the pulse and increasing backwards. The resulting phase modulation causes the frequency of the photons at the front of the pulse to downshift, thus the pulse exhibits a positive chirp. Because the group velocity decreases with decrease in frequency ($v_{\rm {g}}\approx c[1-\omega ^2_{\rm {p}}/2\omega ^2]$), the redshifted spectral components slide backwards, causing the pulse to become asymmetrically compressed^44,45^. As is evident in the figure, the peak magnitude of the field increases to $E_{x}=1.6\,\hbox {TV/m}$ ($a_0\sim 0.4$) after 1.5 cm of propagation.

In the following subsections, we discuss the way the bunch and its qualities evolve with time, and how changing its initial energy, transverse size, density, and the peak power of the laser pulse influence the bunch and its evolution.

### Dynamics of the electron bunch

**Figure 4:**
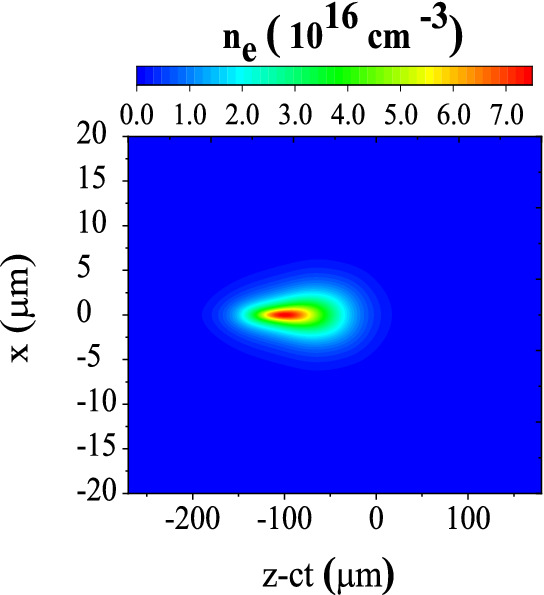
Density distribution of the electron bunch with an initial peak density of $n_{\rm {b}0}=3.5\times 10^{16}\,\mathrm {cm}^{-3}$ at $t=1.4\,\hbox {ps}$.

**Figure 5:**
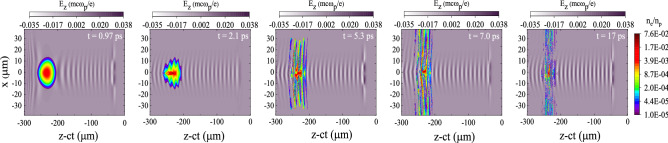
Snapshots showing the accelerating field and the density distribution of the electron bunch with an initial peak density of $n_{\rm {b}0}=3.5\times 10^{16}\,\mathrm {cm}^{-3}$ and an initial energy of 14.8 MeV ($P_{z0}/m_{\rm {e}}c=30$) as a function of transverse position and the speed of light frame coordinate at different propagation times. The peak amplitude of the laser pulse is $a_{0} = 0.25$. The colorbar of the bunch density is logarithmic.

**Figure 6:**
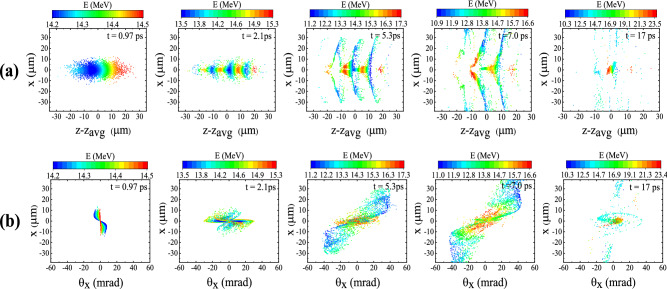
Snapshots showing **(a)** the spatial distribution, and **(b)** the trace-space $\theta _x$–*x* of an electron bunch with an initial energy of 14.8 MeV ($P_{z0}/m_{\rm {e}}c=30$) injected behind a 30 fs laser pulse with $a_{0}$=0.25 at different propagation times.

In order to study the dynamics of an electron bunch in a QPM-LWFA, we analyze its spatial distribution, density evolution, emittance, and energy spectrum as it accelerates in the plasma channel. The density distribution of the bunch at $t=1.4\,\hbox {ps}$, corresponding to an axial distance of $0.42\,\hbox {mm}$, is shown in Fig. 4. The tail of the bunch is compressed in response to the focusing force exerted by its self-field^46–48^. Consequently, the peak density of the bunch is increased to $\sim 7 \times 10^{16}\,\mathrm {cm}^{-3}$, i.e. almost two times its initial value. By “self-field” we mean the wakefield induced in the plasma by the beam itself. Figure 5 displays snapshots of the accelerating field and the density of the electron bunch over an axial distance of 5.2 mm, which is approximately equal to one dephasing length. The laser pulse enters the plasma channel and excites a plasma wave trailing the pulse. As the electrons of the bunch enter the channel, they evolve in response to the wakefield. The length of the electron bunch is longer than the plasma wavelength, therefore the bunch overlaps multiple accelerating and focusing regions and the whole bunch decomposes into many microbunches. The off-axis bunch electrons are either focused or defocused depending on their injection phases. The bunch electrons placed in the focusing areas are focused towards the axis, while the bunch electrons in areas with defocusing fields are pushed away from the axis. The latter electrons experience an increasingly stronger defocusing field as they move outwards further, because quite close to the axis, the transverse focusing/defocusing fields are almost proportional to the distance from the axis. Those electrons which are located in the focusing phase are either accelerated or decelerated by the longitudinal component of the wakefields. The electrons which are in decelerating and focusing fields lose their energy and slip backwards with respect to the wakefield. Consequently, these electrons enter a defocusing region, which results in more electrons being scattered away from the axis (Figs.  and  6a). Figure 6b displays the bunch in the trace space $\theta _x$–*x*. Here, $\theta _x=\tan ^{-1}({P_x}/{P_z})$ is the *x*-dimension inclination angle, in which $P_x$ and $P_z$ are the particle momenta in transverse and axial directions. It shows that the particles moving almost parallel to the axis, i.e. the particles with low $|\theta _x|$, are positioned within $\sim 6\,\mu \hbox {m}$ of the axis. As Fig. 6a indicates, these particles gain the maximum energy. By the way, Fig. 6 is for all electrons, not only the electrons within ROI.

**Figure 7:**
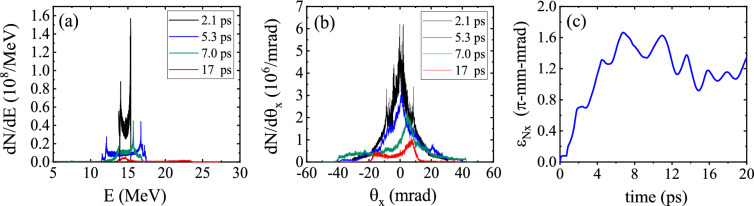
Plots showing how the **(a)** energy spectrum, **(b)** $\theta _x$ distribution, and **(c)** emittance of the bunch change with time.

The energy spectrum of the injected beam at different times is illustrated in Fig. 7a. The energy spectrum is asymmetric because a large fraction of the bunch is initially located in the accelerating phase. The electrons in regions with defocusing fields gradually move to positions far from the axis, where the acceleration is not effective. As a result, the number of electrons accelerated to higher energies decreases with time. As the electrons are continuously accelerated/decelerated in the channel, an increasingly wider range of energies is realized and the energy spread increases. Moreover, due to the fact that the focusing force of the wakefield increases with distance from the axis, the electrons traveling near the axis undergo only very small betatron oscillations and lose little energy, whereas those electrons which are located farther from the axis oscillate with large betatron amplitude and lose a significant amount of energy^49^. This also contributes to the increase in the energy spread of the electron beam. It can be seen that between $t=5.3\,\hbox {ps}$ and $t=7.0\,\hbox {ps}$ the energy spread decreases. Two causes are responsible for this: First, a large portion of the low-energy electrons leave the ROI during this period (Fig. 6a). By $t=10\,\hbox {ps}$ about 80% of the electrons leave the ROI and this number does not change until $t=22\,\hbox {ps}$. Second, the high-energy end of the spectrum shifts towards lower energies due to the deceleration of electrons in that time interval, i.e. between $t=5.3\,\hbox {ps}$ and $t=7.0\,\hbox {ps}$. As shown in Fig. 2a the maximum energy gain decreases between $z=1.6\,\hbox {mm}$ ($t=5.3\,\hbox {ps}$) and $z=2.4\,\hbox {mm}$ ($t=7.8\,\hbox {ps}$). As time progresses, the acceleration/deceleration duality leads to ever higher contrast in terms of electron energy. Thus, in Fig. 7a the highest energy spread happens at $t=17\,\hbox {ps}$. Similary, the angular distribution of the particles spreads out as time passes (Fig. 7b), though because of the much more limited space for acceleration in the perpendicular direction, the spread of the angular distribution is less pronounced compared to the energy spectrum. Figure 7c shows how the emittance of the bunch changes with time. Since the electrons have no initial transverse momentum, the initial emittance is zero. Here, we give a simplified explanation of the variations in emittance. The defocusing fields tend to increase the emittance by pushing the electrons away from the axis. The focusing fields, however, cause the electrons to oscillate transversely around the axis, leading to oscillations in emittance. Furthermore, each time that some group of electrons slip from defocusing fields into focusing fields, the emittance decreases suddenly, provided that the decrease caused by the focusing forces is not compensated completely by the electrons entering defocusing fields. As a result of the combination of these effects, the emittance grows while having some oscillations. As Fig. 2b shows, the amplitude of oscillations in emittance increases considerably after $t\sim 20\,\hbox {ps}$, because around this time a big part of the electron beam leaves focusing field and enters defocusing field. Consequently, some part of it departs ROI, and the remaining part mostly goes to high radial distances, where both focusing and defocusing forces are strong, increasing the amplitude of the oscillations in the emittance and the two standard deviations as well.

### Effect of the initial electron beam energy

**Figure 8:**
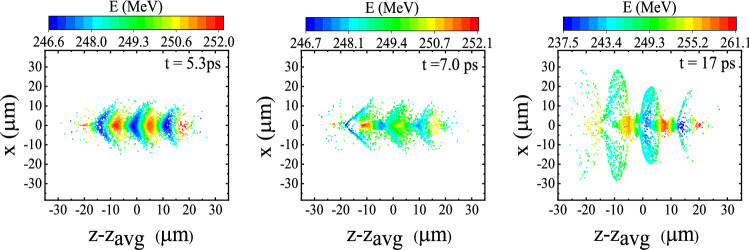
Snapshots of the spatial distribution of an electron beam injected with an initial energy of $E_{0}=249\,\hbox {MeV}$. The other parameters are the same as in Fig. 6a.

To examine the dynamics of the beam at higher initial energy, we consider a monoenergetic bunch with an initial axial momentum of $P_{z0}/m_{\rm {e}}c=500$. The other parameters are the same as above. The comparison of the spatial distribution of the electron bunch for two different initial axial momentums, i.e. $P_{z0}/m_{\rm {e}}c=30$ ($E_0=14.8\,\hbox {MeV}$) and $P_{z0}/m_{\rm {e}}c=500$ ($E_0=249\,\hbox {MeV}$), associated with Figs. 6a and  8, respectively, shows that the bunch electrons remain more collimated in the case of the higher initial energy. Although the maximum energy gain, $\Delta E$, is the same for both situations, higher initial energy leads to lower electron bunch divergence throughout the travel in the channel. Like Figs. 6,  8 is also produced for all bunch electrons, not just the electrons in the ROI.

**Figure 9:**
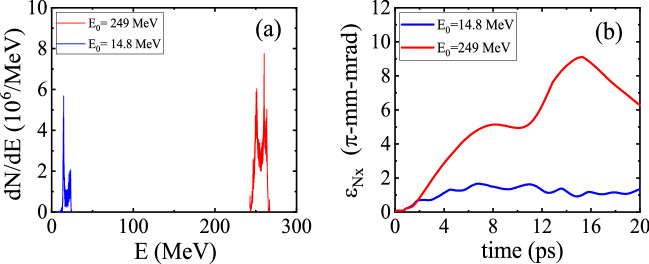
**(a)** Final energy spectra of beam electrons after 17 ps of acceleration, and **(b)** variation of the bunch emittance $\epsilon _{\rm {N},x}$ as a function of the propagation time, for two different initial energies: $E_0=14.8\,\hbox {MeV}$, and $E_0=249\,\hbox {MeV}$.

The corresponding final energy spectra of the electron beam for the two initial energies are illustrated in Fig. 9a. As shown in this figure, the QPM-LWFA generates electron beams with a narrow energy spread. After 5.2 mm of propagation, the energy spreads for $E_0=14.8\,\hbox {MeV}$ and $E_0=249\,\hbox {MeV}$ are $9.4 \%$, and $1.6 \%$, respectively. Since the phase velocity of the $n=-1$ spatial harmonic is set to *c*, the dephasing of the electron bunch with respect to the acceleration field of the $n=-1$ spatial harmonic results in higher energy spread for the beam with lower initial energy. As Fig. 9b indicates, the increase in emittance is higher for the bunch with the greater initial energy. For instance, at a distance of 5.2 mm the normalized rms emittance values are $\epsilon _{\rm {N},x}=1.1\,\pi$-mm-mrad and $\epsilon _{\rm {N},x}=7.7\,\pi$-mm-mrad for $E_0=14.8\,\hbox {MeV}$ and $E_0=249\,\hbox {MeV}$, respectively. This is partly due to the faster growth in the variance of $P_x$ when the initial energy is higher. Furthermore, the betatron oscillation frequency of the electrons is proportional to $\omega _{\beta } \propto \gamma ^{-1/2}$^49^. Therefore, for higher initial energy, the variability in the betatron frequencies of the electrons becomes greater. The spread in the betatron frequencies causes fast betatron phase mixing and bunch decoherence. The bunch decoherence results in an increase in the normalized bunch emittance^49,50^.

### Effect of the laser pulse power

**Figure 10:**
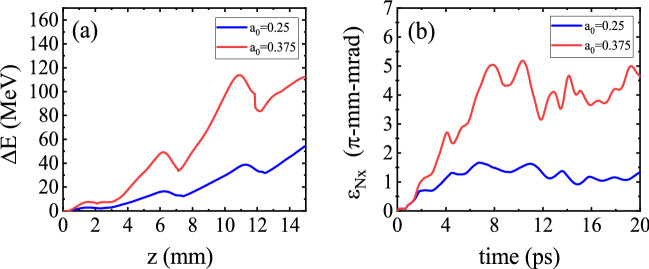
**(a)** Variation of the maximum energy gain $\Delta E$ as a function of distance, and **(b)** bunch emittance $\epsilon _{\rm {N},x}$ as a function of the propagation time for two initial pulse amplitudes of $a_0=0.25$ and $a_0=0.375$. In both cases, the initial energy of electrons is $E_0=14.8\,\hbox {MeV}$.

Since the maximum energy gain in QPM-LWFA scales as $\Delta E \propto a_0^2$, by increasing the laser power $P \propto a_{\rm {0}}^{2}$, higher energy gain can be achieved via the acceleration process. This can be seen in Fig. 10a, in which the maximum energy gain in the corrugated plasma channel is compared for two peak pulse powers, i.e. $P=0.5\,\hbox {TW}$ ($a_0=0.25$) and $P=1.1\,\hbox {TW}$ ($a_0=0.375$). As the transverse field of the $n=-1$ spatial harmonic is proportional to $a_{0}^{2}$^37^, the pulse with higher power produces a stronger wakefield. As a result, a larger fraction of the bunch electrons is defocused. This leads to a greater bunch divergence and the transverse emittance increases, as shown in Fig. 10b. These results indicate that by using a laser pulse of higher power, the transverse properties of the bunch can be degraded despite the increase in the maximum energy gain.

### Effect of the transverse electron bunch size

**Figure 11:**
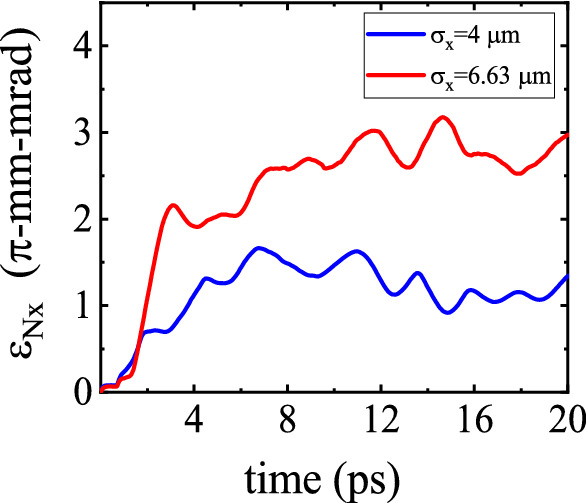
Variation of the bunch emittance $\epsilon _{\rm {N},x}$ as a function of the propagation time for two transverse bunch sizes, $\sigma _x=4\,\upmu \hbox {m}$ and $\sigma _x=6.36\,\upmu \hbox {m}$.

The finite transverse dimensions of the wakefield restrict the effective radial extent for injection. The strength of the focusing/defocusing forces exerted on the off-axis electrons depends on their radial positions. This is due to the fact that, not too far from the axis, the transverse field scales almost linearly with the distance from the axis^37^. Therefore, for the bunch electrons injected in the focusing regions of the wakefield, the higher is the distance of an electron from the axis, the bigger is the amplitude of its betatron oscillations. To investigate the effect of bunch diameter on the transverse properties of the electron bunch, we consider two bunches with different transverse sizes, $\sigma _x=4\,\upmu \hbox {m}$ (corresponding to a bunch diameter of $w_{\rm {b}}=2 \sqrt{2 \ln 2}\,\sigma _x=9.4\,\upmu \hbox {m}$) and $\sigma _x= 6.36\,\upmu \hbox {m}$ ($w_{\rm {b}}=15\,\upmu \hbox {m}$). Both bunches have the same longitudinal size ($\sigma _z=8\,\upmu \hbox {m}$) and total charge (11 pC). In Fig. 11, the evolution of transverse emittance is compared for these two bunches. For the bunch with the higher diameter, the transverse emittance, $\epsilon _{\rm {N},x}$, increases more rapidly owing to the combination of two causes: the variance of the radial positions is larger simply because the initial transverse beam size is larger, and the bunch electrons experience stronger transverse forces on the whole, because the average distance of the electrons from the axis is larger.

### Effect of the bunch density

The externally injected electron bunch moving through the plasma channel can be affected by both the laser-driven wakefield and the wakefield originated from the interaction of the beam itself with the plasma. The wakefield deformation along the bunch caused by beam loading can impinge on the dynamics and final parameters of the beam. However, controlling beam loading can increase the performance of the accelerator.

**Figure 12:**
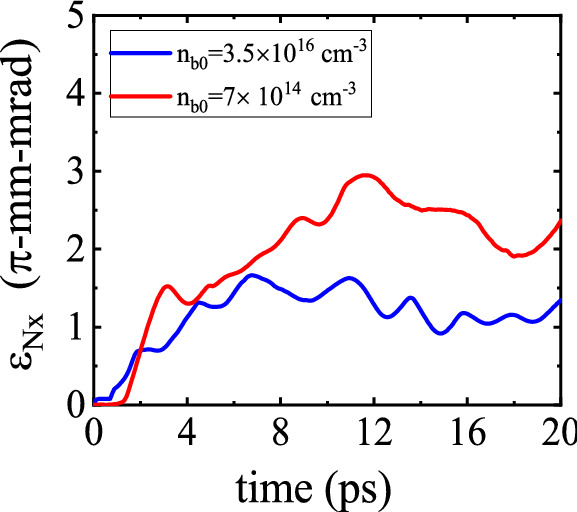
Variation of the bunch emittance $\epsilon _{\rm {N},x}$ as a function of the propagation time for two beam densities, $n_{\rm {b}0}=3.5 \times 10^{16}\,\mathrm {cm}^{-3}$ and $n_{\rm {b}0}=7 \times 10^{14}\,\mathrm {cm}^{-3}$.

In this subsection, the influence of the initial bunch density on the transverse emittance is investigated briefly. Figure 12 shows the time evolution of the normalized transverse emittance for peak beam densities of $n_{\rm {b}0}=3.5 \times 10^{16}\,\mathrm {cm}^{-3}$ and $n_{\rm {b}0}=7 \times 10^{14}\,\mathrm {cm}^{-3}$. This figure indicates that the collimation and final quality of the beam can be improved by increasing the bunch density. At the higher beam density, the space charge force of the beam blows out the channel electrons more strongly and the resulting field decreases the divergence of the beam.

## Discussion

In the present paper, using 2D PIC simulations, the dynamics of externally injected electron bunches traveling in a QPM-LWFA are studied. The energy spectra exhibit quasi-monoenergetic peaks with energy spreads which depend on the initial energy of the electron beam. At higher initial energy, some portions of the bunch remain in phase with the longitudinal components of the wakefield over a longer distance and the energy spread is lower. Moreover, increasing the initial energy of the electron beam can impede the scattering of the bunch. In our simulations, the emittance growth for the higher initial energy is 7 times of magnitude larger than for the lower initial bunch energy. Higher pulse power can increase the maximum energy gain, but it increases the final transverse emittance of the bunch, $\epsilon _{\rm {N},x}$, as well. For bunches with larger transverse diameters, the stronger transverse wakefield felt at higher radial positions, together with the larger variance of the radial coordinate, leads to an increase in $\epsilon _{\rm {N},x}$. Therefore, injection of a bunch with lower diameter can improve the bunch emittance. Increasing the beam density results in improved collimation of the electrons due to the favorable effect of beam loading on the transverse component of the wakefield. The defocusing of the electrons can be mitigated by using the proper bunch density.

## Methods

### Particle-in-cell simulations

Simulation results shown in the paper were performed with the particle-in-cell code OSIRIS^43^. We consider the linear regime of electron acceleration. A 30 fs (FWHM), 0.8 $\upmu$m, 0.5 TW laser pulse with a sine-squared temporal profile and a Gaussian transverse profile is focused into a waist radius of $w_0=15\,\upmu \hbox {m}$. The magnitude of the normalized vector potential is $a_{0} = 0.25$. The target plasma density was ramped up over 200 $\upmu$m. After the initial ramp, the plasma density followed corrugated channel (Eq. 1). The values of the parameters for the density profile are $n_{\rm {p0}}=7 \times 10^{18}\,\mathrm {cm}^{-3}$, $\delta =0.04$, and $\lambda _{\rm {m}}=5\,\hbox {mm}$. The parameters of the electron beam are: $\sigma _{x}=4\,\mu \hbox {m}$ and $\sigma _{z}=8\,\upmu \hbox {m}$, and $n_{\rm {b0}}=3.5 \times 10^{16}\,\mathrm {cm}^{-3}$, where $n_{b0}$ is the electron beam peak density. The total electron charge is about 11 pC and it is initially located $200\,\upmu \hbox {m}$ behind the peak of the laser pulse, coinciding with the peak of the moving envelope of the $n=-1$ spatial harmonic. The initial energy of the electrons is 14.8 MeV. The simulation window moves at the speed of light (*c*) along the direction of propagation and has dimensions of 438 $\upmu$m $\times$ 77 $\upmu$m. The number of grid points is 16384 $\times$ 512. Four macro-particles per cell are considered for the plasma and 9 macro-particles for the beam. Absorbing boundaries for the laser field and all of the particle species are defined around the simulation box.

## Data Availability

The data that supports the results of this study is available from the corresponding author upon reasonable request.
